# The associations between different sleep patterns and osteoporosis based on the Osteoporosis Self-Assessment Tool for Asians

**DOI:** 10.1007/s11657-020-00828-y

**Published:** 2020-10-17

**Authors:** Shaojun Wu, Pengbo Wang, Xiaofan Guo, Guozhe Sun, Ying Zhou, Zhao Li, Hongmei Yang, Shasha Yu, Liqiang Zheng, Yingxian Sun

**Affiliations:** 1 Department of Cardiology, The First Hospital of China Medical University, 155 Nanjing North Street, Heping District, Shenyang, 110001 Liaoning Province People’s Republic of China; 2 Department of Clinical Epidemiology, Library, Shengjing Hospital of China Medical University, Shenyang, 110001 Liaoning Province People’s Republic of China

**Keywords:** Sleep duration, Daytime nap, Osteoporosis, Osteoporosis Self-Assessment Tool for Asians (OSTA)

## Abstract

***Summary*:**

Based on the use of Osteoporosis Self-Assessment Tool for Asians (OSTA) to assess osteoporosis risk, we found that short sleep duration and taking a daytime nap had an increased risk of osteoporosis.

**Purpose:**

To explore the associations between different sleep patterns with osteoporosis.

**Methods:**

3659 postmenopausal women (average age of 60 years) were divided into low, middle, and high osteoporosis risk categories based on the Osteoporosis Self-Assessment Tool for Asians (OSTA). After having collected by a standard questionnaire, total and nocturnal sleep duration was collapsed to form categories of ≤ 6 h, > 6 h and ≤ 7 h, > 7 h and ≤ 8 h, > 8 h and ≤ 9 h, > 9 h, and daytime nap duration of 0 h and > 0 h.

**Results:**

As a categorical variable, the total sleep duration of ≤ 6 h per day (OR = 1.34, 95% CI 1.04–1.72), nocturnal sleep duration of ≤ 6 h per night (OR = 1.65, 95% CI 1.24–2.18), and taking a daytime nap (OR = 1.33, 95% CI 1.09–1.64) had higher osteoporosis risk after adjustment for covariates. As a continuous variable, after the adjustment for covariates, both longer total (OR = 0.86, 95% CI 0.78–0.94) and nocturnal sleep duration (OR = 0.83, 95% CI 0.76–0.91) had lower risk of osteoporosis risk while taking longer daytime nap (OR = 1.10, 95% CI 1.02–1.19) had higher osteoporosis risk.

**Conclusions:**

Postmenopausal women with both short total and nocturnal sleep duration (6 h or less) and taking a daytime nap had increased osteoporosis risk as assessed by OSTA.

## Introduction

Osteoporosis is a systemic bone disease characterized by low bone mineral density (BMD) and micro-architectural deterioration of bone tissue that affects 200 million people worldwide [1]. The commonly unrecognized fracture caused by osteoporosis is often associated with increased mortality, morbidity [2], and health costs [3]. Gender, height, lifestyle patterns, vitamin D intake, and some other potential variables are widely recognized as the risk factors for osteoporosis [4].

Sleep, a fundamental physiological activity and closely related to circadian rhythms, was found to be involved with bone metabolism [5–7]. In addition, a series of epidemiological studies had been performed to explore the association between sleep duration and osteoporosis, of which some [8–10] stated that long sleep duration (8 h or more per day) might be associated with a higher risk of osteoporosis and some [11–13] suggested that those with short sleep duration were more likely to have higher risk of osteoporosis. Interestingly, Swanson et al. [14] reported that between sleep duration and osteoporosis risk in older postmenopausal women, there was no significant association.

Daytime nap, accompanied by many diseases such as obstructive sleep apnea [15] and neuropsychiatric disorders [16], was widely accepted by many Chinese to make up for the short of sleep at night. However, it seemed that taking a daytime nap has been associated with higher risk of mortality [17] while the associations between daytime naps and osteoporosis risk was seldom studied.

Besides the few number of related studies and inconsistent findings, we also noticed that some of these studies were focused on postmenopausal women and used common tools to assess osteoporosis like quantitative ultrasound (QUS); however, it is the tools they used that were not specific for evaluating osteoporosis risk in postmenopausal women. Thus, in the present study, we firstly tried to use the Osteoporosis Self-Assessment Tool for Asians (OSTA), which is specific for Asian postmenopausal women’s osteoporosis risk assessment [4], to further explore the association between different sleep patterns and osteoporosis in Chinese postmenopausal women.

## Methods and materials

### Study population

This cross-sectional study was part of the Northeast China Rural Cardiovascular Health Study (NCRCHS) From January 2013 to August 2013, which enrolled 14,016 permanent residents who were older than 35 years of age with 11,956 (85.3%) completed the study. The participants were selected randomly from three regions (Dawa, Zhangwu, and Liaoyang) of Liaoning Province. In the present study, women were included if they had a natural menopause but not a surgical or medically induced menopause for more than 1 year [18]. Then, the participants were excluded if they had missing necessary sleep information, reasons for poor sleep (such as some psychological or neurological disorders), or taken any drugs affecting sleep or bone metabolism during the last 14 days. In the end, a total of 3659 individuals were available for this cross-sectional study.

Written informed consent was obtained from all participants or their proxies if the participants were illiterate, and the study was approved by the Ethics Committee of China Medical University in Shenyang, China.

### Osteoporosis assessment

The Osteoporosis Self-Assessment Tool for Asians (OSTA) was developed by Koh et al. [4], an index which was initially generated by recruiting Asian postmenopausal women in eight Asian countries or regions. After having evaluated the potential risk factors for osteoporosis and validated in Japan, the OSTA index equation, 0.2 × [body weight (kg) − age (years)], proved to perform well for identifying postmenopausal women with osteoporosis. In addition, two studies [19, 20] reported superior combined sensitivity and specificity with the OSTA index, compared with the US Preventive Services Task Force (USPSTF). Another study [21] indicated that to select men older than 70 years for osteoporosis screening, the OSTA index performed even better than the more complex Fracture Risk Assessment Tool (FRAX). Therefore, in our study, we calculated the osteoporosis risk index using the OSTA index equation and classified the participants into low (< − 4), middle (− 4 to − 1), and high (> − 1) osteoporosis risk categories according to the recommended criterion.

### Sleep patterns

Information on individual sleep patterns including total sleep duration, nocturnal sleep duration, and daytime nap duration was collected by a standard questionnaire. According to total and nocturnal sleep duration, the participants were classified into five groups: ≤ 6 h, > 6 h and ≤ 7 h, > 7 h and ≤ 8 h, > 8 h and ≤ 9 h, and > 9 h, of which > 7 h and ≤ 8 h was selected as the reference category because it was recommended for adults [22]. Two groups of 0 h and > 0 h were set up at the same time by daytime nap duration and the former was the reference category.

### Other measurements and definitions

In the present study, demographics and menopause status were collected at the research visit by experienced cardiologists and trained nurses who used a standard questionnaire to conduct a face-to-face interview. Waist circumference and height were measured both to the nearest 0.1 cm, weight was measured to the nearest 0.1 kg, then the waist to height ratio (WHtR) was calculated as waist circumference (cm) divided by height (cm), and BMI was calculated as the individual’s weight (kg) divided by the square of the height (m^2^). Fasting blood samples were obtained in the morning after more than 8 h of fasting for all participants. Blood samples were collected from an antecubital vein into vacutainer tubes containing EDTA. Serum was subsequently isolated from whole blood and all serum samples were frozen at − 20 °C for testing at a central, certified laboratory. Total cholesterol (TC), triglycerides (TG), high-density lipid cholesterol (HDL-C), and low-density lipid cholesterol (LDL-C) were measured with an auto-analyzer. All data were recorded by standard methods and all laboratory equipment was calibrated.

All participants were asked about the current status of smoking, drinking, taking estrogen, and the history of hyperthyroidism. Three times (45 min/time) a week for at least 3 months exercise was considered a regular physical activity. Educational level was divided into three levels: low (primary school or less), middle (middle school and high school), and high (college or more). Hypertension was defined as a systolic blood pressure (SBP) ≥ 140 mmHg, a diastolic blood pressure (DBP) ≥ 90 mmHg, and/or receiving treatment for hypertension; diabetes mellitus was diagnosed as a fasting blood glucose (FBG) ≥ 7 mmol/L and/or receiving anti-diabetic treatment.

### Statistical analysis

Baseline characteristics were presented as mean ± standard deviation or frequencies with percentages, compared by *P* values from a trend test. The mean duration of different sleep patterns among osteoporosis risk categories were calculated and presented; *P* values from a trend test was used to evaluate the associations between them. To test the associations between osteoporosis risk and total sleep duration, nocturnal sleep duration, or daytime nap duration categories, two logistic regression models were established. Model A was unadjusted and the potential risk factors for osteoporosis were further adjusted in Model B. The odds ratios (ORs) and 95% confidence intervals (CIs) were shown.

SPSS 25.0 for Windows (SPSS Inc., Chicago, USA) was used for analysis, *P* < 0.05 was considered significant.

## Results

Baseline characteristics of the 3659 postmenopausal women at the average age of 60 years across osteoporosis risk groups are presented in Table 1. The low osteoporosis risk occurred in 2329 (63.7%) participants, middle osteoporosis risk in 1007 (27.5%), and high osteoporosis risk in 323 (8.8%). The mean age (*P* < 0.001) and HDL-C (*P* < 0.001) increased while the mean BMI (*P* < 0.001), WHtR (*P* < 0.001), TG (*P* < 0.001), and LDL-C (*P* = 0.001) decreased across the osteoporosis risk categories. Hypertension, currently smoking, currently drinking, and regular physical activity were more often found in individuals with high osteoporosis risk (65.9%, 33.7%, 5.6%, and 30.0%, respectively) while those who were diagnosed with diabetes mellitus were prone to low osteoporosis risk. Furthermore, we found most of the participants were Han nationality (*P* = 0.001) with a low level of education (*P* < 0.001). When it comes to currently taking estrogen (*P* = 0.821), history of hyperthyroidism (*P* = 0.914), and TC (*P* = 0.359), there were no significant associations with the osteoporosis risk. Last but not least, those who had short total (*P* = 0.001) and nocturnal (*P* < 0.001) sleep duration (6 h or less) and taking a daytime nap (*P* < 0.001) were prone to high osteoporosis risk.

**Table 1:** Baseline characteristics of study participants across osteoporosis risk categories

Characteristics	Total (*n* = 3659)	Osteoporosis risk			*P* for trend
		Low (*n* = 2329)	Middle (*n* = 1007)	High (*n* = 323)	
Age, years	60 ± 8	56 ± 6	64 ± 6	73 ± 6	< 0.001
Age at menopause, years	49 ± 5	49 ± 5	49 ± 5	48 ± 4	0.029
BMI, kg/m^2^	24.83 ± 3.86	26.33 ± 3.51	22.71 ± 2.84	20.69 ± 2.86	< 0.001
WHtR	0.53 ± 0.07	0.55 ± 0.06	0.51 ± 0.06	0.50 ± 0.07	< 0.001
Education level, *n* (%)					
Low	2647 (72.3)	1532 (65.8)	837 (83.1)	278 (86)	< 0.001
Middle	957 (26.1)	758 (32.5)	165 (16.4)	44 (13.6)	
High	32 (0.87)	31 (1.33)	1 (0.09)	0 (0.00)	
Nation, *n* (%)					0.001
Han	3487 (95.3)	2198 (94.4)	976 (96.9)	313 (96.9)	
Other	172 (4.7)	131 (5.6)	31 (3.1)	10 (3.1)	
Currently smoking, *n* (%)					
Yes	739 (20.2)	357 (15.3)	273 (27.1)	109 (33.7)	< 0.001
No	2920 (79.8)	1972 (84.7)	734 (72.9)	214 (66.3)	
Currently drinking, *n* (%)					
Yes	107 (2.9)	54 (2.3)	35 (3.5)	18 (5.6)	0.001
No	3552 (97.1)	2275 (97.7)	972 (96.5)	305 (94.4)	
Currently taking estrogen, *n* (%)					
Yes	32 (0.9)	20 (0.9)	10 (1.0)	2 (0.6)	0.821
No	2792 (76.3)	1836 (78.8)	760 (75.5)	196 (60.7)	
Regular physical activity, *n* (%)					
Yes	973 (26.6)	596 (25.6)	280 (27.8)	97 (30.0)	0.048
No	2686 (73.4)	1733 (74.4)	727 (72.2)	226 (70.0)	
Hypertension, *n* (%)					
Yes	2131 (58.3)	1339 (57.5)	579 (57.6)	213 (65.9)	0.028
No	1527 (41.7)	990 (42.5)	427 (42.4)	110 (34.1)	
Diabetes mellitus, *n* (%)					0.004
Yes	491 (13.5)	338 (14.6)	123 (12.3)	30 (9.3)	
No	3148 (86.5)	1981 (85.4)	875 (87.7)	292 (90.7)	
History of hyperthyroidism, *n* (%)					
Yes	96 (2.6)	61 (2.6)	30 (3.0)	5 (1.5)	0.914
No	2892 (79.0)	1910 (82.0)	779 (77.4)	203 (62.8)	
Total sleep duration, h					
≤ 6, *n* (%)	1456 (39.8)	868 (37.3)	444 (44.1)	144 (44.6)	0.001
> 6 and ≤ 7, *n* (%)	709 (19.4)	467 (20.1)	197 (19.6)	45 (13.9)	
> 7 and ≤ 8, *n* (%)	866 (23.7)	576 (24.7)	225 (22.3)	65 (20.1)	
> 8 and ≤ 9, *n* (%)	410 (11.2)	270 (11.6)	95 (9.4)	45 (13.9)	
> 9, *n* (%)	199 (5.4)	135 (5.8)	42 (4.2)	22 (6.8)	
Nocturnal sleep duration, h					
≤ 6, *n* (%)	981 (26.8)	554 (23.8)	310 (30.8)	117 (36.2)	< 0.001
> 6 and ≤ 7, *n* (%)	799 (21.8)	505 (21.7)	236 (23.4)	58 (18.0)	
> 7 and ≤ 8, *n* (%)	704 (19.2)	466 (20.0)	193 (19.2)	45 (13.9)	
> 8 and ≤ 9, *n* (%)	934 (25.5)	643 (27.6)	211 (21.0)	80 (24.8)	
> 9, *n* (%)	232 (6.3)	155 (6.7)	54 (5.4)	23 (7.1)	
Daytime nap duration, h					
= 0, *n* (%)	2487 (68.0)	1661 (71.3)	653 (64.8)	173 (53.6)	< 0.001
> 0, *n* (%)	1158 (31.6)	659 (28.3)	351 (34.9)	148 (45.8)	
TG, mmol/L	1.82 ± 1.46	1.93 ± 1.59	1.64 ± 1.19	1.55 ± 1.13	< 0.001
TC, mmol/L	5.60 ± 1.12	5.61 ± 1.15	5.61 ± 1.09	5.54 ± 0.98	0.359
LDL-C, mmol/L	3.18 ± 0.86	3.21 ± 0.33	3.15 ± 0.85	3.05 ± 0.78	0.001
HDL-C, mmol/L	1.42 ± 0.35	1.38 ± 0.33	1.46 ± 0.37	1.54 ± 0.40	< 0.001

*BMI*, body mass index; *WHtR*, waist to height ratio; *TG*, triacylglycerol; *TC*, cholesterol; *LDL-C*, low-density lipoprotein cholesterol; *HDL-C*, high-density lipoprotein cholesterol

**Fig. 1:**
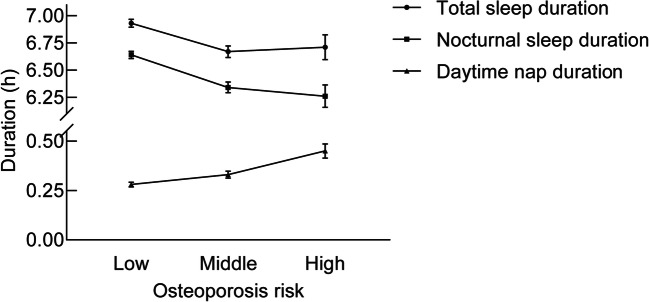
The mean durations of different sleep patterns by osteoporosis risk categories

The mean duration of different sleep patterns by osteoporosis risk category is presented in Fig. 1. As a result, the mean duration of nocturnal sleep duration decreased significantly with advancing osteoporosis risk category from the highest of 6.64 h to the lowest of 6.26 h (*P* < 0.001). The opposite trend was observed in daytime nap duration with the mean value increased from the lowest of 0.28 h to the highest of 0.45 h (*P* < 0.001). Individuals with low osteoporosis risk were more likely to have the longest total sleep duration while those in middle osteoporosis risk category had the shortest (*P* < 0.001).

Unadjusted and multivariable adjusted ORs and 95% CIs for osteoporosis risk are presented in Table 2 according to baseline total sleep duration, nocturnal sleep duration, and daytime nap duration, expressed as categorical variable respectively. A 1.34-fold risk of osteoporosis in Model A (OR = 1.34, 95% CI 1.13–1.60) and a 1.35-fold risk of osteoporosis in Model B (OR = 1.35, 95% CI 1.04–1.72) were detected in total sleep duration of 6 h or less per day, compared with total sleep duration of > 7 h and ≤ 8 h. Nocturnal sleep duration of ≤ 6 h was also more likely to have higher osteoporosis risk, compared with that of > 7 h and ≤ 8 h both in Model A (OR = 1.55, 95% CI 1.28–1.89) and Model B (OR = 1.65, 95% CI 1.24–2.18). Participants taking a nap (daytime nap duration > 0 h) were prone to higher osteoporosis risk (OR = 1.58, 95% CI 1.37–1.81), compared with those who had no daytime nap (daytime nap duration = 0 h), and after adjustment for covariates, this trend persisted (OR = 1.33, 95% CI 1.09–1.64). There were no significant associations of other total sleep duration, nocturnal sleep duration, and daytime nap duration categories with osteoporosis risk when compared with the corresponding reference category.

**Table 2:** Logistic regression models for osteoporosis risk and different sleep patterns (as categorical variable)

Variables	Number	Model A^a^		Model B^b^	
		OR (95% CI)	*P* value	OR (95% CI)	*P* value
Total sleep duration, h					
≤ 6	1456	1.34 (1.13,1.60)	0.001	1.35 (1.04,1.72)	0.021
> 6 and ≤ 7	709	1.01 (0.82,1.24)	0.933	0.89 (0.67,1.21)	0.470
> 7 and ≤ 8	866	1 (reference)	–	1 (reference)	–
> 8 and ≤ 9	410	1.08 (0.85, 1.38)	0.528	1.04 (0.73, 1.47)	0.828
> 9	199	0.99 (0.72, 1.38)	0.992	0.84 (0.51, 1.36)	0.485
Test of parallel lines	–	–	0.111	–	0.191
Nocturnal sleep duration, h					
≤ 6	981	1.55 (1.28, 1.89)	< 0.001	1.65 (1.24, 2.18)	0.001
> 6 and ≤ 7	799	1.14 (0.92, 1.40)	0.231	1.29 (0.96, 1.74)	0.094
> 7 and ≤ 8	704	1 (reference)	–	1 (reference)	–
> 8 and ≤ 9	934	0.92 (0.75, 1.14)	0.460	1.13 (0.84, 1.58)	0.429
> 9	232	1.03 (0.75, 1.39)	0.865	1.13 (0.72, 1.77)	0.590
Test of parallel lines	–	–	0.121	–	0.187
Daytime nap duration, h					
= 0	2487	1 (reference)	–	1 (reference)	–
> 0	1158	1.58 (1.37, 1.81)	< 0.001	1.33 (1.09, 1.64)	0.006
Test of parallel lines	–	–	0.145	–	0.137

^a^Model A was unadjusted

^b^Model B was adjusted for age at menopause, BMI, WHtR, education level, nation, currently smoking, currently drinking, currently taking estrogen, physical activity, hypertension, diabetes mellitus, history of hyperthyroidism, TG, TC, LDL-C, and HDL-C

Unadjusted and multivariable adjusted ORs and 95% CIs for osteoporosis risk are also presented in Table 3 according to different sleep patterns, but expressed as continuous variable. Both in Model A (per 1 SD: OR = 0.87, 95% CI 0.82–0.93) and Model B (per 1 SD: OR = 0.86, 95% CI 0.78–0.94), longer total sleep duration was significantly associated with a lower risk of osteoporosis risk. In addition, getting more nocturnal sleep was also more likely to have lower osteoporosis risk whether in Model A (per 1 SD: OR = 0.82, 95% CI 0.76–0.87) or Model B (per 1 SD: OR = 0.83, 95% CI 0.76–0.91). Interestingly, individuals taking longer daytime nap were prone to higher osteoporosis risk (per 1 SD: OR = 1.16, 95% CI 1.09–1.24), and the same trend was observed even after adjustment for covariates (per 1 SD: OR = 1.10, 95% CI 1.02–1.19).

**Table 3:** Logistic regression models for osteoporosis risk and different sleep patterns (as continuous variable)

Variables	Mean ± SD	Model A^a^		Model B^b^	
		OR (95% CI)	*P* value	OR (95% CI)	*P* value
Total sleep duration					
Per 1 SD	0.000 ± 1.000	0.87 (0.82, 0.93)	< 0.001	0.86 (0.78, 0.94)	0.001
Test of parallel lines	–	–	0.232	–	0.145
Nocturnal sleep duration					
Per 1 SD	− 0.001 ± 1.000	0.82 (0.76, 0.87)	< 0.001	0.83 (0.76, 0.91)	< 0.001
Test of parallel lines	–	–	0.618	–	0.148
Daytime nap duration					
Per 1 SD	− 0.002 ± 0.997	1.16 (1.09, 1.24)	< 0.001	1.10 (1.02, 1.19)	0.017
Test of parallel lines	–	–	0.155	–	0.291

*SD*, standard deviation

^a^Model A was unadjusted

^b^Model B was adjusted for age at menopause, BMI, WHtR, education level, nation, currently smoking, currently drinking, currently taking estrogen, physical activity, hypertension, diabetes mellitus, history of hyperthyroidism, TG, TC, LDL-C, and HDL-C

## Discussion

In the present cross-sectional study, we found a significant association between different sleep patterns and osteoporosis even after adjustment for the potential confounding factors based on the Osteoporosis Self-Assessment Tool for Asian postmenopausal women to assess osteoporosis risk. Both total sleep duration and nocturnal sleep duration of 6 h or less per day were related to an increased risk of osteoporosis when compared with total sleep duration or nocturnal sleep duration of > 7 h and ≤ 8 h, and compared with those taking no daytime nap (daytime nap duration = 0 h), individuals taking a nap (daytime nap duration > 0 h) were significantly associated with an elevated osteoporosis risk.

Inadequate sleep duration (< 6 h) was associated with osteoporosis. A latest research of 11,084 women in America reported that short nocturnal sleep duration was associated with a higher risk of osteoporosis when comparing across the different sleep duration categories [11]. Another Chinese study also indicated that an increased risk of osteoporosis in women with nocturnal sleep duration of 7 h or less per night was detected [12]. In addition, Fu et al. [13] found that compared with those who slept 8 h, women at the age of 18 to 80 years who slept 6 h or less per night had a significantly lower level of BMD and a high level of osteoporosis risk. These three cross-sectional studies support our findings that short sleep duration may be associated with an increased risk of osteoporosis.

On the contrary, long sleep was also associated with osteoporosis. A Japanese study that included 19,321 individuals older than 50 years stated that self-reported sleep duration of more than 8 h have higher odds of osteoporosis [8]. Another Chinese sample that included 31,769 participants (aged 45–86 years) also found that long sleeping ≥ 8 h was more likely to have higher osteoporosis risk [9]. The present work did not agree with findings of the two cross-sectional studies, which may be due to the fact that we focused on postmenopausal women, but the Japanese study included more than 50% of men and few participants in the Chinese sample (men for 5.0%, women for 4.9%) reported sleeping 7 h or less hours per night.

To our knowledge, even though the underlying mechanism between sleep duration and osteoporosis risk is not fully understood, the decrease in melatonin [23] and growth hormone (GH) [24], and the increase in glucocorticoids [25] and inflammatory cytokines [26, 27] resulted from inadequate sleep which could play an important role. First, a randomized controlled trial (RCT) [28] found that treatment with melatonin increased BMD while the same result was observed in an animal study [29], another RCT [30] indicated that GH treatment in postmenopausal osteoporosis reduced the fracture incidence by 28% during the 10 years follow-up, and Tritos’ analysis [31] also suggested that GH treatment was associated with decreased fracture risk in adults. Then, two studies [32, 33] presented that elevated circulating glucocorticoids reduced the osteoblast function while the two other studies [34, 35] demonstrated that increased circulating inflammatory cytokines had the same effect. To sum up, inadequate sleep could disrupt endocrine and metabolic function, leading to the decreased bone mineral density and osteoblast formation, thus resulting in an increased osteoporosis risk.

It was also a noteworthy finding that compared with those taking no daytime nap, individuals taking a nap were significantly associated with an elevated risk of osteoporosis in the present study. Similar result was found in previous studies of Stone et al. [36] and Chen et al. [37]. One potential explanation is that taking a daytime nap is trying to compensate for poor nocturnal sleep [37]. As we demonstrated in Table 3, longer daytime nap duration was significantly associated with a greater risk of osteoporosis with an odds ratio of 1.10 per standard deviation while longer nocturnal sleep duration was significantly associated with a lower risk of osteoporosis with an odds ratio of 0.83 per standard deviation in ♦adjusted analyses. Another explanation could be the significant decrease in 6-sulfatoxymelatonin level [38] and urinary melatonin level [39] during daytime sleep among women.

There were several strengths in the present study. First, sleep duration was divided into five categories, which allowed us to evaluate the association between total or nocturnal sleep duration and osteoporosis more thoroughly. Second, a number of potential risk factors for osteoporosis were included in the analysis, such as age at menopause, smoking, and drinking. Third, we explored the associations between osteoporosis and different sleep patterns by firstly using the OSTA index which was developed specifically for Asian postmenopausal women to assess osteoporosis risk.

However, some limitations also exist in the present study. First, this is a cross-sectional study; therefore, although short total sleep duration, short nocturnal sleep duration, and taking a daytime napping were associated with higher osteoporosis risk, we could not deduce the causal associations between them. Second, data about some factors affecting bone metabolism, such as previous fragility fractures for the women, history of rheumatoid arthritis, and family history of hip fractures, calcium and vitamin D intakes were not collected in the present study. Third, information on sleep patterns were collected by the use of self-reported measures like many studies, which might cause potential recall bias.

In conclusion, the present study found that significant associations existed between different sleep patterns and osteoporosis risk. Both total sleep duration and nocturnal sleep duration of 6 h or less per day were related to an increased risk of osteoporosis when compared with total sleep duration or nocturnal sleep duration of > 7 h and ≤ 8 h, and individuals taking a nap were significantly associated with an elevated risk of osteoporosis compared with those taking no daytime nap. However, further researches related to the topic including more complete and objective information are needed to be performed.
